# Motile sperm organelle morphology examination (MSOME): intervariation study of normal sperm and sperm with large nuclear vacuoles

**DOI:** 10.1186/1477-7827-8-56

**Published:** 2010-06-07

**Authors:** João Batista A Oliveira, Claudia G Petersen, Fabiana C Massaro, Ricardo LR Baruffi, Ana L Mauri, Liliane FI Silva, Juliana Ricci, José G Franco

**Affiliations:** 1 Department of Gynecology and Obstetrics, Botucatu Medical School São Paulo State University - UNESP, Botucatu, Brazil; 2 Paulista Center for Diagnosis, Research and Training, Ribeirao Preto - SP, Brazil; 3 Center for Human Reproduction Prof Franco Jr., Ribeirao Preto, Brazil; 4 Postgraduate Fellow Department of Gynecology and Obstetrics, Botucatu Medical School Sao Paulo State University -- UNESP, Brazil

## Abstract

**Background:**

Although the motile sperm organelle morphology examination (MSOME) was developed only as a selection criterion, its application as a method for classifying sperm morphology may represent an improvement in evaluation of semen quality, with potential clinical repercussions. The present study aimed to evaluate individual variations in the motile sperm organelle morphology examination (MSOME) analysis after a time interval.

**Methods:**

Two semen samples were obtained from 240 men from an unselected group of couples undergoing infertility investigation and treatment. Mean time interval between the two semen evaluations was 119 +/- 102 days. No clinical or surgical treatment was realized between the two observations. Spermatozoa were analyzed at greater than or equal to 8400× magnification by inverted microscope equipped with DIC/Nomarski differential interference contrast optics. At least 200 motile spermatozoa per semen sample were evaluated and percentages of normal spermatozoa and spermatozoa with large nuclear vacuoles (LNV/one or more vacuoles occupying >50% of the sperm nuclear area) were determined. A spermatozoon was classified as morphologically normal when it exhibited a normal nucleus (smooth, symmetric and oval nucleus, width 3.28 +/- 0.20 μm, length 4.75 +/- 0.20 μm/absence of vacuoles occupying >4% of nuclear area) as well as acrosome, post-acrosomal lamina, neck and tail, besides not presenting cytoplasm around the head. One examiner, blinded to subject identity, performed the entire study.

**Results:**

Mean percentages of morphologically normal and LNV spermatozoa were identical in the two MSOME analyses (1.6 +/- 2.2% vs. 1.6 +/- 2.1% *P *= 0.83 and 25.2 +/- 19.2% vs. 26.1 +/- 19.0% *P *= 0.31, respectively). Regression analysis between the two samples revealed significant positive correlation for morphologically normal and for LNV spermatozoa (r = 0.57 95% CI:0.47-0.65 *P *< 0.0001 and r = 0.50 95% CI:0.38-0.58 *P *< 0.0001, respectively).

**Conclusions:**

The significant positive correlation and absence of differences between two sperm samples evaluated after a time interval with respect to normal morphology and LNV spermatozoa indicated that MSOME seems reliable (at least for these two specific sperm forms) for analyzing semen. The present result supports the future use of MSOME as a routine method for semen analysis.

## Background

To test the hypothesis that subtle sperm organelle malformations could be associated with the ICSI result, Bartoov *et al. *[1] developed a new method for real-time evaluation of sperm morphology, the motile sperm organelle morphology examination (MSOME). MSOME is accomplished by utilizing an inverted light microscope equipped with high-power Nomarski optics enhanced by digital imaging to achieve a magnification above 6000×, much higher than the magnification used habitually by embryologists in sperm selection for the ICSI procedure (200× to 400×) or even the level employed in routine semen examination (1000×). Recent studies have demonstrated that intracytoplasmic morphologically selected sperm injection, based on sperm normality as defined by MSOME, significantly improves fertilization rate [2,3], embryo quality [3-6], development rate up to the blastocyst stage [3,7], rates of implantation and pregnancy after transference on day 2 or 3 [2,4-6,8-10] or in the blastocyst stage [7,11] and the chance of having a healthy normal child [12], while the miscarriage rate was significantly decreased [4,5,8-10].

Although MSOME was developed only as a selection criterion, as demonstrated in studies, its application as a method for classifying sperm morphology may represent an improvement in the evaluation of semen quality, with potential clinical repercussions, particularly with regard to assisted reproduction techniques [7,13-15]. To comprehend the diagnostic/prognostic value, the present study aimed to evaluate the within-subject variation of seminal morphology evaluated by MSOME analysis after a time interval.

## Methods

### Study participants

Two semen samples were obtained from 240 men from an unselected group of couples undergoing infertility investigation and treatment. The average age was 38.0 ± 5.7 years (range: 27-55 years); 31.7% (76/240) had fathered at least one child (or a pregnancy that had ended in miscarriage); 14.2% (34/240) had varicocele; 10.4% (25/240) were smokers; 47.9% (115/240) regularly used alcohol. The mean duration of infertility was 4.3 ± 3.2 years (range: 1-19 years). Male infertility was present in 44.2% (106/240) of the couples. Written informed consent was obtained from all the men on the day of first semen sample collection. This study received internal institutional review board approval.

### Sample collection

Semen samples were collected in sterile containers by masturbation after a sexual abstinence period of 2-5 days. No clinical or surgical treatment was realized between the two observations. During the study none of the men experienced febrile illness. The semen sample was immediately taken and processed for MSOME. The liquefied fresh semen samples were prepared by Isolate (Irvine Scientific, USA) discontinuous concentration gradient. The final pellet was resuspended in 0.2 ml modified human tubal fluid (HTF) medium (Irvine Scientific) and then sent for MSOME.

### Determination of morphology by MSOME

An aliquot of 1 μl of sperm cell suspension was transferred to a 5 μl microdroplet of modified HTF medium containing 7% polyvinylpyrrolidone solution (PVP medium; Irvine Scientific). This microdroplet was placed in a sterile glass dish (FluoroDish; Word Precision Instrument, USA) under sterile paraffin oil (Ovoil-100; VitroLife, Goteborg, Sweden). The sperm cells, suspended in the microdroplet, were placed on a microscope stage above an Uplan Apo ×100 oil/1.35 objective lens previously covered by a droplet of immersion oil. In this manner, suspended motile sperm cells in the observation droplet could be examined at high magnification by an inverted microscope (Eclipse TE 2000U; Nikon, Japan) equipped with high-power differential interference contrast optics (DIC/Nomarski). The images were captured by a color video camera containing effective picture elements (pixel) for high quality image production, and a color video monitor. Morphological evaluation was accomplished on a monitor screen and the total calculated magnification was ≥8400× (total magnification: objective magnification × magnification selector × video coupler magnification × calculated video magnification).

Two forms of spermatozoa observed at MSOME were considered in this study: normal spermatozoa and spermatozoa with large nuclear vacuoles (LNV). A spermatozoon was classified as morphologically normal when it exhibited a normal nucleus as well as acrosome, post-acrosomal lamina, neck and tail, besides not presenting cytoplasm around the head [1]. The subcellular organelles were morphologically classified on the basis of the presence of specific malformations, which were defined according to the arbitrary descriptive approach reported by Bartoov et al. [1] after studies utilizing transmission and scanning electron microscopy: acrosome: absent, partial or vesiculated; post-acrosomal lamina: absent or vesiculated; neck: abaxial, disordered or showing cytoplasmic droplet; tail: absent, coiled, broken, multi or short.

**Figure 1:**
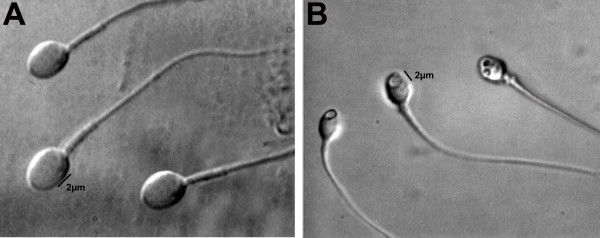
**Morphological sperm forms**. **A: **Normal spermatozoa observed at high magnification (≥8400×); **B: **Spermatozoa with large nuclear vacuoles observed at high magnification (≥8400×).

For the nucleus, also according to transmission electron microscopy estimations [1,8], the morphological normal state was defined by the shape and content of the chromatin. The criterion for normality of nuclear shape was a smooth, symmetric and oval configuration. Normal means for length and width were estimated as 4.75 ± 2.8 and 3.28 ± 0.20 μm [1] respectively, where the form classified as abnormal presented variation of 2SD on at least one of the axes (length: ≥5.31 or ≤4.19 μm, width: >3.7 or <2.9 μm). For rapid evaluation of nuclear shape, a fixed, transparent, celluloid form of sperm nucleus fitting the criteria was superimposed on the examined cell (chablon construction based on ASTM E 1951-2[16]). The criterion for normality of chromatin content was the absence of vacuoles occupying >4% of the sperm nuclear area. Figure 1A shows normal spermatozoa analyzed by MSOME.

LNV spermatozoa were defined (Bartoov modified classification) by the presence of one or more vacuoles occupying >50% of the sperm nuclear area (visual evaluation aided, if necessary, by a celluloid form of a large vacuole superimposed on the examined cell). Figure 1B shows LNV spermatozoa analyzed by MSOME.

The same technician performed all sperm selection. As done in other sperm morphological analyses, each sperm was evaluated/classified individually in MSOME, a process carried out directly on the monitor screen (no pictures were taken). At least 200 motile spermatozoa per sample were evaluated and the percentage of normal and LNV spermatozoa were determined. The analysis lasted 30--60 min/sample (≈same time for sperm selection using MSOME).

### Quality control

To determine intra-technician variability, fractions of motile spermatozoa were obtained from randomly selected patients. Each sample was observed at least three times by the same observer. A variation of 0.5% was obtained for all parameters analyzed: normality of the spermatozoon as a whole, normality of nuclear form, normality of chromatin, spermatozoon with nuclear vacuoles as a whole, and spermatozoa with vacuoles occupying >50% of the nuclear area. The inter-observer variability was not evaluated because only one observer, blinded to subject identity, performed the entire study.

### Statistical analysis

Data were analyzed using InStat version 3.0 (GraphPad Software, San Diego, CA, USA) on a Macintosh computer (Apple Computer Inc., Cupertino, CA, USA). The Wilcoxon matched-pairs signed-ranks test and logistic regression were used. Correlations were assessed via the Spearman rank correlation test. Normal form and LNV percentages by MSOME at 1^st ^and 2^nd ^exams were treated as a continuous variable for analysis. The significance level was set at *P *< 0.05.

## Results

Logistic regression did not show association between normal sperm forms at MSOME and the subgroups of men involved in at least one pregnancy (odds Ratio (OR) = 1.00; 95% Confidence Interval (CI) = 0.88 to 1.13), with varicocele (OR= 0.82; 95% CI: 0.64 to 1.04), smokers (OR = 0.93; 95% CI = 0.74 to 1.15) or regular alcohol users (OR = 0.93; 95% CI = 0.835 to 1.05). Equally, logistic regression also did not show an association between sperm forms with vacuoles occupying >50% of the nuclear area and the subgroups of men involved in at least one pregnancy (OR= 0.98; 95% CI = 0.97 to 1.00), smokers (OR = 1.01; 95% CI = 0.99 to 1.03) or regular alcohol users (OR = 1.00; 95% CI = 0.99 to 1.01). However, men with varicocele presented more spermatozoa with LNV (OR = 1.02; 95% CI = 1.00 to 1.03)

**Figure 2:**
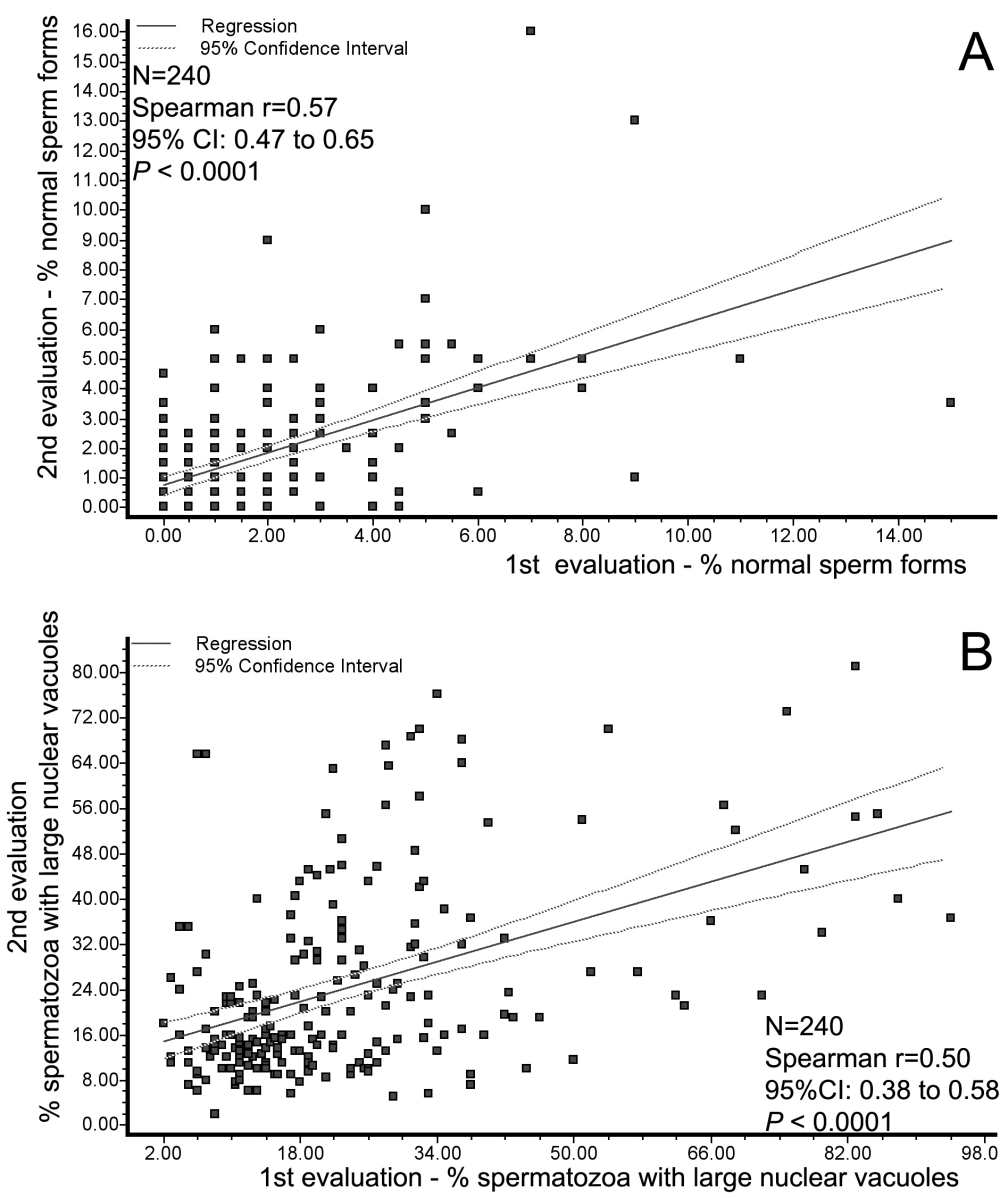
**1° and 2° evaluation -Correlations**. **A: **Relationship between percentages of normal sperm forms at first (x) and second evaluation (y). Individual data points, regression line and confidence interval (CI) are shown. Spearman rank correlation coefficient = 0.57; *P *< 0.0001; **B: **Relationship between percentage of large-nuclear-vacuole forms at first (x) and second (y) evaluation. Individual data points, regression line and confidence interval (CI) are shown. Spearman rank correlation coefficient = 0.50; *P *< 0.0001.

Regression analysis demonstrated significant positive correlation between percentage of normal sperm forms between the first and second evaluation by MSOME (*P *< 0.0001; Spearman's rank correlation coefficient, *r *= 0.57; 95% confidence interval: 0.47-0.65). In relation to LNV forms, regression analysis also demonstrated significant positive correlation between percentage at the first and second exam (*P *< 0.0001; Spearman's rank correlation coefficient, *r *= 0.50; 95% confidence interval: 0.38-0.58). Figure 2 summarizes this result.

**Figure 3:**
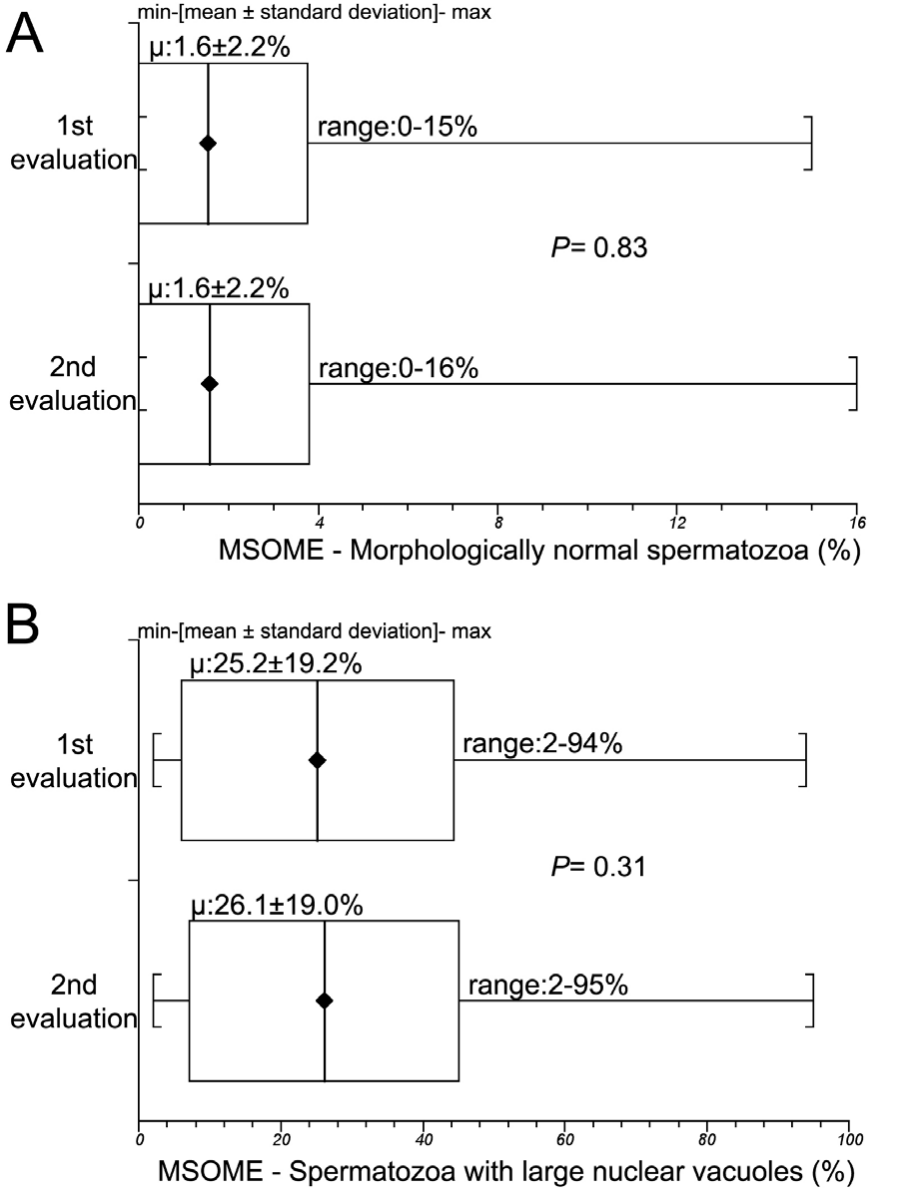
**1° and 2° evaluation -- Incidences**. **A: **Morphologically normal sperm incidences did not differ statistically between the two MSOME evaluations (*P *= 0.83). **B: **Incidences of large-nuclear-vacuole sperm did not differ statistically between the two MSOME evaluations (*P *= 0.31).

The mean incidence of morphologically normal spermatozoa did not differ statistically (*P *= 0.83) between the first (1.6 ± 2.2%, range: 0--15%) and second samples examined (1.6 ± 2.2, range: 0-16%). Similarly, in the MSOME exams the LVN sperm incidence did not differ significantly (*P *= 0.31) between first (25.2 ± 19.2%, range:2--94%) and second samples (26.1 ± 19.0, range:2-95%). Figure 3 summarizes these findings.

The mean interval between the two MSOME evaluations was 119 ± 102days. Comparing the incidence of normal and LNV sperm forms in relation to the time interval (≤60days, >60-≤120days, >120-≤180days, >180days) did not evidence a significant difference. Table 1 summarizes these results.

**Table 1:** Incidence of morphologically normal and large-nuclear-vacuole spermatozoa in two MSOME evaluations according to patient subgroup time interval

Subgroups	MSOME		
	1^st ^evaluation	2^nd ^evaluation	*P*
Interval			
≤60 days (n:79)			
Normal	1.7 ± 2.5%	1.8 ± 2.1%	ns
	n:3.4 ± 5	n:3.7 ± 4.1	ns
LNV	23.7 ± 17.7%	24.6 ± 17.1%	ns
	n:47.4 ± 35.3	n:49.2 ± 34.3	ns
>60-≤120 days (n:85)			
Normal	1.2 ± 1.5%	1.4 ± 2.2%	ns
	n:2.4 ± 3.0	n:2.8 ± 4.5	ns
LNV	26.3 ± 18.3%	27.6 ± 19.2%	ns
	n:52.7 ± 36.7	n:55.2 ± 38.4	ns
>120-≤180days (n:32)			
Normal	1.4 ± 1.8%	1.4 ± 2.2%	ns
	n:2.9 ± 3.7	n:2.2 ± 2.9	ns
LNV	24.4 ± 20.2%	27.6 ± 19.2%	ns
	n:48.9 ± 40.5	n:41.6 ± 33.7	ns
>180 days (n:44)			
Normal	2.0 ± 2.9%	1.7 ± 2.7%	ns
	n:4.0 ± 5.9	n:3.5 ± 5.6	ns
LNV	26.1 ± 22.9%	29.6 ± 22.4%	ns
	n:52.2 ± 45.8	59.3 ± 44.9	ns

ns = not statistically significant

## Discussion

The accuracy with which morphological normality of spermatozoa can be assessed depends on the resolution power of the optical magnification system. Spermatozoa appearing as morphologically normal at 1000× magnification may in fact carry various structural abnormalities that can only be detected at higher optical magnifications (>6000×). The improvement in observation is mainly due to the replacement of Hoffman modulation contrast by the Nomarski interferential modulation contrast. Thus, the use of MSOME may represent, potentially, improvement in morphological analysis of sperm.

Although semen analysis remains fundamental to the evaluation of male fertility, heterogeneities described between semen samples from the same individual may undermine the diagnostic reliability of a single exam. In fact, different studies [17,18] highlighted marked interejaculate coefficients of variation for normal morphology. However, our data showed, besides the significant positive correlation found between the two MSOME evaluations for both morphological sperm forms analyzed, lack of variations in the mean percentages of normal sperm (P = 0.83) or sperm with LNV (*P *= 0.31). In fact, the MSOME classification system was shown to be very stable, presenting mean percentages of normal sperm (1.6 ± 2.2% vs. 1.6 ± 2.2%) and LNV sperm (25.2 ± 19.2% vs 26.1 ± 19.0) that were practically identical between the two evaluations. It was particularly interesting that variations between the two morphological MSOME evaluations were also not observed even when the interval between the exams was much more than 10 weeks, a period of one spermatogenic cycle [19], reinforcing the stable aspect of the analysis.

On the other hand, as the percentage of normal spermatozoa determined by MSOME was low (1.6%) in this study, the random counting variation of only 200 spermatozoa would be huge relative to the normal form percentage. So, this large counting variation could lead to absence of difference between samples. However, two considerations must be taken into account. First, it is important to note that the frequency of normal sperm forms in our results was not very different from those reported in previous studies, in general <4% of the mean (1,15). This low percentage probably occurred because MSOME is a much stricter criterion of sperm morphology classification. In addition, the percentages of sperm with LNV, which were higher than percentages of normal spermatozoa (25-26%), were also shown to be stable. Thus, the number of spermatozoa evaluated per sample apparently did not significantly impair the evaluation of the percentages.

Unfortunately MSOME application beyond sperm selection is not usual. In fact, to the best of our knowledge, the present study was the first that analyzed intra-individual variation of the MSOME and thus cannot be compared with other results. However, our data are in agreement with other studies that used others morphological sperm evaluation criteria. Employing recommendations of the WHO Manual [20], Oshio et al. [21] and Gao et al. [22] found high individual agreement between different morphological evaluations. Using the Tygerberg criterion [23], Smit et al. [24] and Mishail et al. [25] did not observe statistically significant intra-individual fluctuations in sperm morphology.

In our results, the MSOME normal sperm forms appear to be uninfluenced by previous involvement in at least one pregnancy, varicocele or the regular use of tobacco or alcohol. Similarly, the LNV forms did not present significant statistical differences among the majority of male subgroups, with the exception of the varicocele group. However, a large additional semen analysis is necessary to draw a final conclusion given the low frequencies of the some subgroups, and the low percentages of normal sperm forms.

The choice in analyzing the LNV sperm in this study was motivated by the clinical implications. Bartoov et al. [1,26] and Berkovitz et al. [8], based on electron microscopy data, assumed that nuclear vacuoles indicate chromatin abnormality. Other studies confirmed the association between nuclear vacuoles at high magnification and chromatin damage. Berkovitz *et al.*, [9] graded the severity of nuclear morphological alterations, highlighting principally the presence of large vacuoles and suggesting that vacuolization of the sperm nucleus reflects some underlying chromosomal or DNA defect. Franco *at al*. [27] demonstrated an association between large nuclear vacuoles and both the presence of DNA fragmentation and denaturation in the spermatozoa. Garolla et al. [28] showed that the presence of nuclear vacuoles affects mitochondrial function, chromatin status, and aneuploidy rate. Toshimori and Ito [29], using electron microscopy, associated the presence and content of nuclear vacuoles with DNA damage. In addition, the authors emphasize that IMSI/MSOME aids in identifying vacuoles. Oliveira et al. [30] observed a significant positive correlation between the percentage of sperm that present large nuclear vacuoles and the percentage of DNA fragmentation. On the other hand, the resolution power offered by MSOME enables inclusion of spermatozoa with intranuclear vacuoles that would not be detected in the conventional evaluation. Thus, based on clinical/laboratory findings on the repercussions of possible DNA damage for offspring [31], the stability and reliability of identification of sperm nuclear vacuoles by MSOME observed in our results can represent improvement in morphological sperm evaluation influencing, consequently, the therapeutic decision. In fact, our data agree with recent studies that propose classifications for defining semen quality based on analysis at high magnification, with special emphasis on the number and extension of nuclear vacuoles [7,13,32].

## Conclusions

In conclusion, the present results demonstrate significant positive correlation and absence of differences between two sperm samples evaluated after a time interval with respect to normal morphology and LNV spermatozoa, thus indicating that MSOME seems to be a stable method (at least for these two specific sperm forms) for analyzing semen. The present result supports the future use of MSOME as a routine method for semen analysis.

## Competing interests

The authors declare that they have no competing interests.

## Authors' contributions

JBAO was responsible for designing and coordinating the study. All authors were responsible for data collection, data analysis, and data interpretation presented in the manuscript. JBAO, RLRB and JGF were responsible for the statistical work and for writing of the manuscript. JGF was responsible for reviewing the manuscript. All authors read and approved the final manuscript.
